# Cardiac MRI stress testing in the reduction of radiation exposure for patients undergoing ischemic evaluation

**DOI:** 10.1186/1532-429X-18-S1-P195

**Published:** 2016-01-27

**Authors:** Daniel Morgenstern, Brandon M Mikolich, Nicola B Nicoloff, J Ronald Mikolich

**Affiliations:** 1 Sharon Regional Health System, Hermitage, PA USA; 2 Northeast Ohio Medical University, Rootstown, OH USA

## Background

Cardiac diagnostic testing is responsible for a significant portion of non-therapeutic radiation exposure in the United States. The major source of cardiac-related radiation exposure is nuclear based myocardial perfusion imaging (MPI). Furthermore, a higher radio-isotope dose is necessary for MPI in obese patients, a growing segment of the population. As of January 1, 2014, the American Society of Nuclear Cardiology (ASNC) recommended that measures be taken to ensure a mean dose of ≤9.0 mSv per MPI study, a goal which has been difficult for many institutions to achieve. This study was designed to assess the role of cardiac MRI (CMR) stress imaging in reducing radiation exposure in a "real-world" setting, particularly the growing subset of obese patients.

## Methods

An institutional cardiac imaging database was queried for all patients who underwent a MPI or CMR stress imaging procedure for evaluation of ischemic heart disease in 2014. In anticipation of the ASNC mean dose guidelines, an institutional recommendation (not policy) was implemented for patients with a BMI > 35 to preferentially undergo CMR stress testing. The records of all stress study patients were available for review after IRB approval, and the radiation dose of each patient was recorded, allowing computation of the mean radiation dose for this cohort of patients.

## Results

2,413 patients underwent MPI or CMR stress, 1,830 with a BMI < 35 and 583 with a BMI > 35. If all of these patients had a nuclear MPI, the mean radiation dose would have been 17.45 mSv. As a result of the CMR institutional recommendation, 277 patients underwent a CMR stress instead, lowering the mean radiation dose to 14.9 mSv, a 16% reduction. There were 399 patients with a BMI > 35 who still had a nuclear MPI, despite the institutional recommendation. If these patients had undergone a stress CMR, the mean radiation dose would have dropped to 11.3 mSv, realizing a 36% potential reduction. To reach the ASNC goal of ≤ 9.0 mSv per study, 55% of the remaining 1,737 patients with a BMI < 35 would be required to have a "stress only" MPI study.

## Conclusions

CMR stress is capable of reducing the mean institutional radiation dose for nuclear MPI by circumventing the higher isotope doses required for patients with a BMI > 35. The radiation dose reduction is 14% in a real-world setting, but policy driven mandatory CMR stress for patients with a BMI > 35, could potentially result in a 40.2% reduction in mean radiation dose. Use of CMR stress for obese patients cannot lower the mean radiation dose to less than or equal to 9.0 mSv, because of the inherent radiation exposure in stress-rest MPI protocols. After use of CMR stress for obese patients, approximately 55% of nuclear MPI stress patients would require a "stress-only" MPI study to meet current ASNC goals for controlling radiation dose in evaluation of ischemic patients.

